# Supplementary material for: Functionalized Au15 nanoclusters as luminescent probes for protein carbonylation detection
Source: Commun Chem. 2021 May 14;4:69. doi: 10.1038/s42004-021-00497-z (PMC9814629; doi:10.1038/s42004-021-00497-z)
Supplement: Supplementary file 4 — Description of Additional Supplementary Files [file 42004_2021_497_MOESM4_ESM.pdf]

Description of Additional Supplementary Files

File Name: Supplementary Data 1

Description:

- Supplementary Figure S5: Original gels for Fig. 3b
- Supplementary Figure S6: Replicate gels #2 for Fig. 3b
- Supplementary Figure S7: Replicate gels #2 for Fig. 3b
- Supplementary Figure S8: Original blots and replicates for Supplementary Fig. 3a
- Supplementary Figure S9: Original gels for Fig. 4a
- Supplementary Figure S10: Replicate gels #2 for Fig. 4a
- Supplementary Figure S11: Replicate gels #3 for Fig. 4a
- Supplementary Figure S12: Original scan for Fig. 4b
- Supplementary Figure S13: Original gels for Supplementary Fig. S4

File Name: Supplementary Data 2

Description:

- Supplementary Figure S14: Raw data obtained after quantification of blots from Supplementary figure S8 using image lab software.
- Supplementary Figure S15: Raw data from the colorimetric assay in Supplementary figure S3b
