# Supplementary material for: Functionalized Au15 nanoclusters as luminescent probes for protein carbonylation detection
Source: Commun Chem. 2021 May 14;4:69. doi: 10.1038/s42004-021-00497-z (PMC9814629; doi:10.1038/s42004-021-00497-z)
Supplement: Supplementary file 5 — Supplementary Information [file 42004_2021_497_MOESM5_ESM.pdf]

Supplementary information for

Functionalized Au_{15} nanoclusters as luminescent probes for protein carbonylation detection

Guillaume F. Combes,[1,2] Hussein Fakhouri,[1,3] Christophe Moulin,[3] Marion Girod,[4] Franck Bertorelle,[3] Srestha Basu,[3] Romain Ladouce,[2] Martina Perić Bakulić,[1] Željka Sanader Maršić,[5] Isabelle Russier-Antoine,[3] Pierre-François Brevet,[3] Philippe Dugourd,[3] Anita Krisko,[6] Katarina Trajković,[1,2] Miroslav Radman,[1,2,7] Vlasta Bonačić-Koutecký[1,8,9,✉] and Rodolphe Antoine[3,✉]

[1]*Center of Excellence for Science and Technology-Integration of Mediterranean Region (STIM), Faculty of Science, University of Split, Ruđera Boškovića 33, 21000 Split, Croatia*

[2]*Mediterranean Institute for Life Sciences (MedILS), 21000 Split, Croatia*

[3]*Institut Lumière Matière, UMR5306, Université Claude Bernard Lyon1-CNRS, Univ. Lyon 69622, Villeurbanne cedex, France*

[4]*Univ Lyon, Université Claude Bernard Lyon 1, CNRS, Institut des Sciences Analytiques, UMR5280, F-69622, Lyon, France*

[5]*Faculty of Science, University of Split, Rudera Boskovica 33, 21000 Split, Republic of Croatia*

[6]*Department of Experimental Neurodegeneration, University Medical Center Goettingen, Germany*

[7]*Université R. Descartes-Paris 5, Faculté de Médecine, site Cochin, 75014 Paris, France*

[8]*Interdisciplinary Center for Advanced Science and Technology (ICAST) at University of Split, Meštrovićevo šetalište 45, 21000 Split, Croatia*

[9]*Chemistry Department, Humboldt University of Berlin, Brook-Taylor-Strasse 2, 12489 Berlin, Germany*

[✉]*email: vbk@cms.hu-berlin.de; rodolphe.antoine@univ-lyon1.fr*

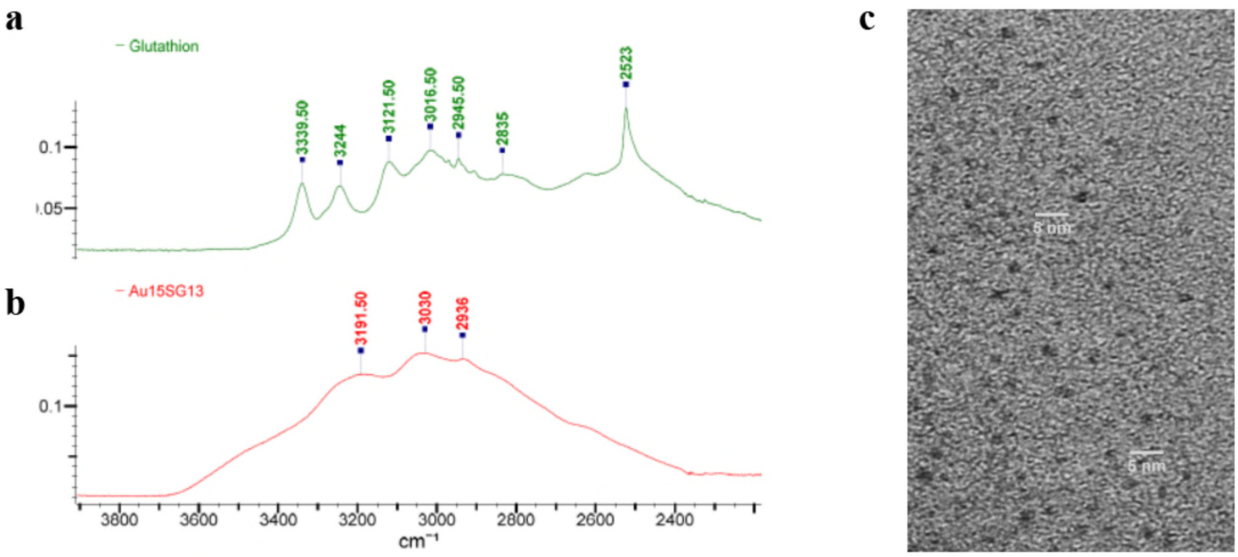

<u>Supplementary Figure S1:</u> Characterization of Au_{15} NCs a.-b. FTIR spectra of $Au_{15}SG_{13}$ NCs and pure GSH. The peak at 2523 cm^{-1}, which corresponds to S-H stretching vibration mode, disappears in the $Au_{15}SG_{13}$ NCs. **c.** TEM image of the $Au_{15}SG_{13}$ NCs.

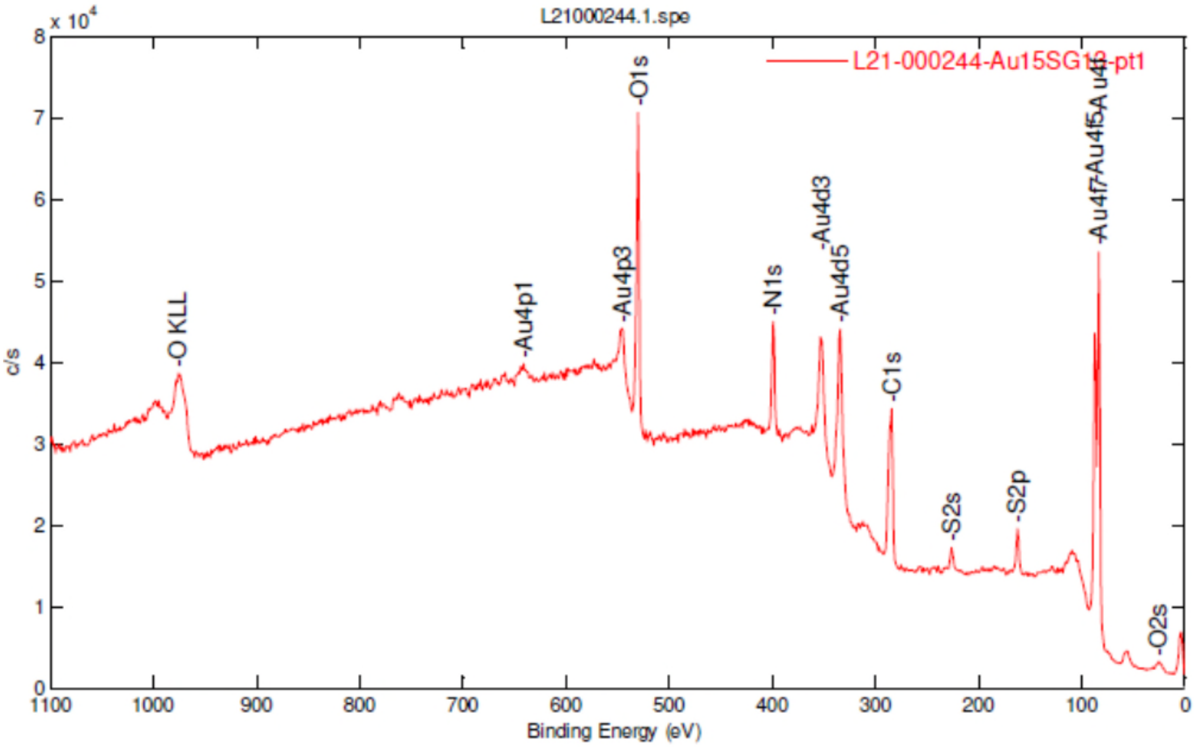

	C	O	N	S	Au
$Au_{15}SG_{13}$ clusters (% content)	44.3	30.6	15.1	4.3	5.4

<u>Supplementary Table S1:</u> Elemental and quantitative XPS analysis of $Au_{15}SG_{13}$ NCs.

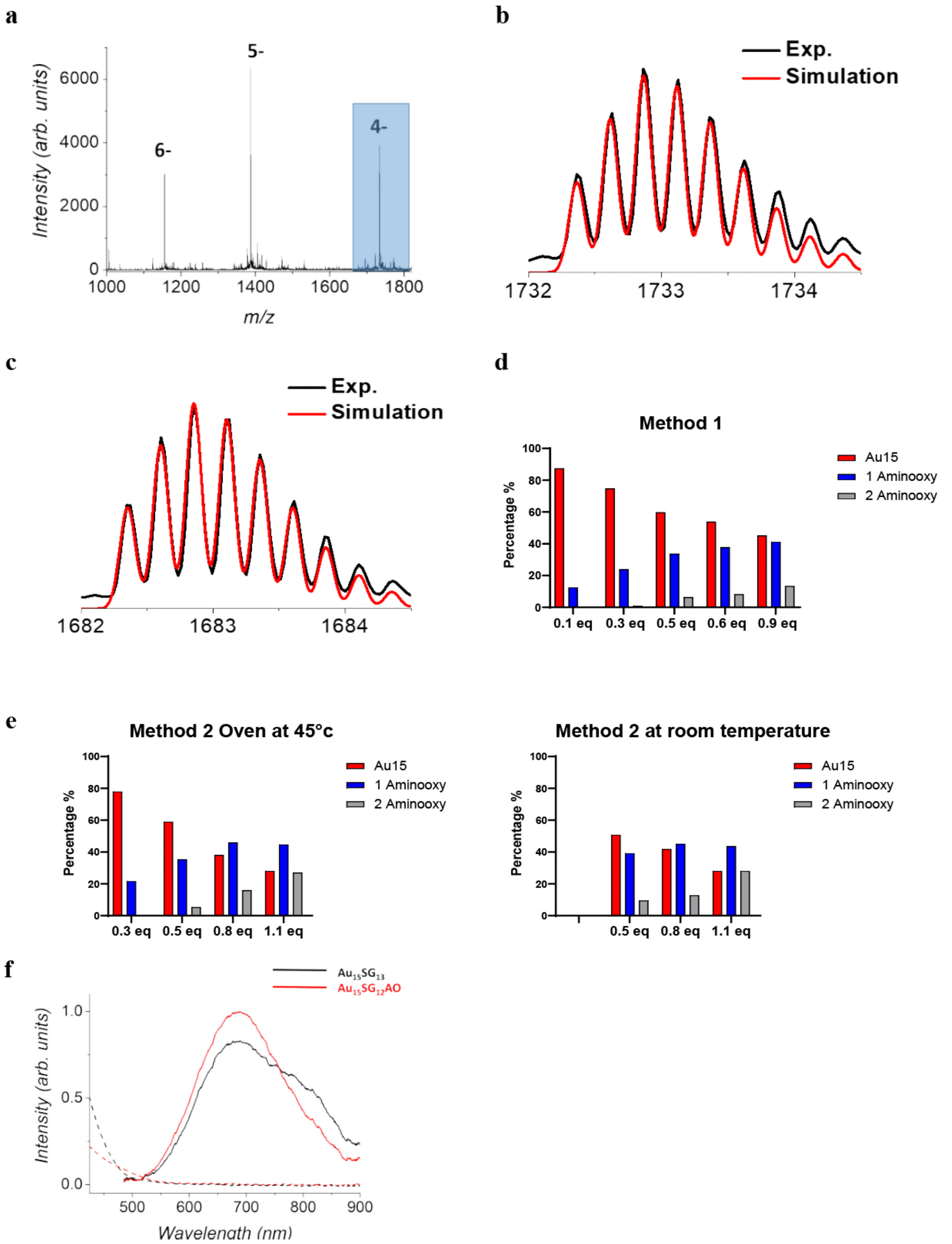
a
Intensity (arb. units)
6000
4000
2000
0
6-
5-
4-
1000
1200
1400
1600
1800
m/z
b
Exp.
Simulation
1732
1733
1734
c
Exp.
Simulation
1682
1683
1684
d
Method 1
Percentage %
100
80
60
40
20
0
0.1 eq
0.3 eq
0.5 eq
0.6 eq
0.9 eq
Au15
1 Aminooxy
2 Aminooxy
e
Method 2 Oven at 45°c
Percentage %
100
80
60
40
20
0
0.3 eq
0.5 eq
0.8 eq
1.1 eq
Au15
1 Aminooxy
2 Aminooxy
Method 2 at room temperature
Percentage %
100
80
60
40
20
0
0.5 eq
0.8 eq
1.1 eq
Au15
1 Aminooxy
2 Aminooxy
f
$Au_{15}SG_{13}$
$Au_{15}SG_{12}AO$
Intensity (arb. units)
1.0
0.5
0.0
500
600
700
800
900
Wavelength (nm)

g

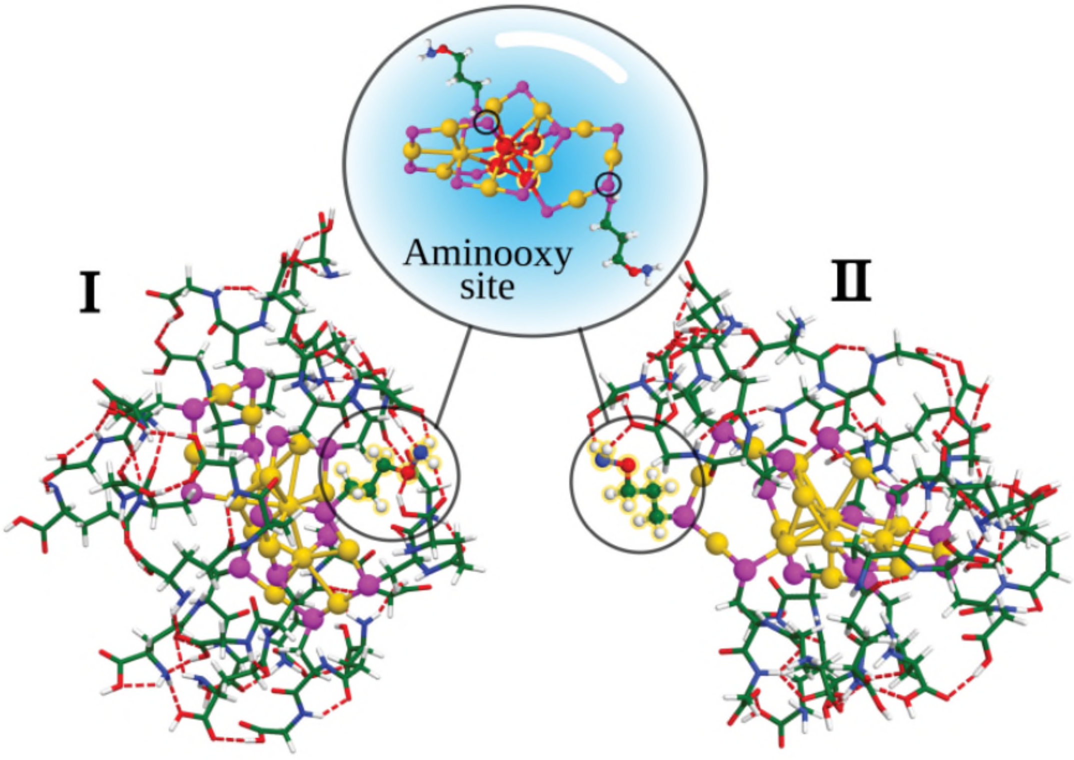

Supplementary Figure S2: Characterization and modeling of Au_{15} NCs a. ESI mass spectrum of $Au_{15}SG_{13}$ NCs dispersed in water. A charge state distribution from $[M-4H]^{4-}$ (4-) through $[M-6H]^{6-}$ (6-) is observed. **b.-c.** Isotopic patterns of the different clusters (4- charge states of **b.** $Au_{15}SG_{13}$ and **c.** $Au_{15}SG_{12}$-Ao) are in perfect agreement with the simulated ones. **d.-e.** Graphs showing the percentage of different compounds after adding AO using 2 ligand exchange methods **d.** method 1 and **e.** method 2 as describe in the method section. **f.** UV-vis absorption spectra (dashed lines) and fluorescence spectra (straight lines) (with excitation at 473 nm) the synthesized AuNCs. **g.** The structures for isomers I and II $Au_{15}SG_{12}$-Ao have been obtained using DFT/PM7 approach as well as by DFT(B3LYP) single point calculations. In isomer I Ao site is bound to the NC core taking central position while in isomer II Ao site takes peripheral position participating within the staple motif. The energy difference between I and II ΔE=0.86 eV was obtained by single point DFT(B3LYP) approach. Atoms are labelled as follows: S-magenta, Au-gold, C-green, N-blue, O-red, H-white. Hydrogen bonds are labelled by red dots.

a

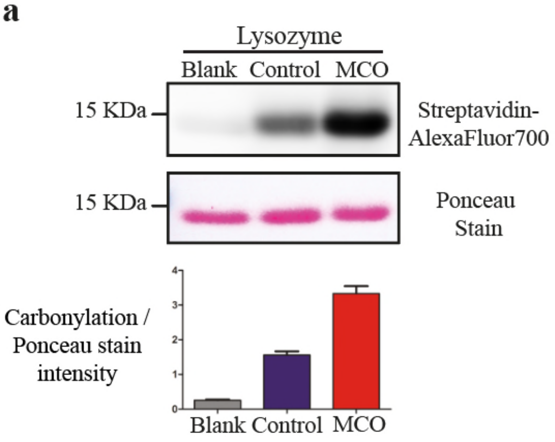

b

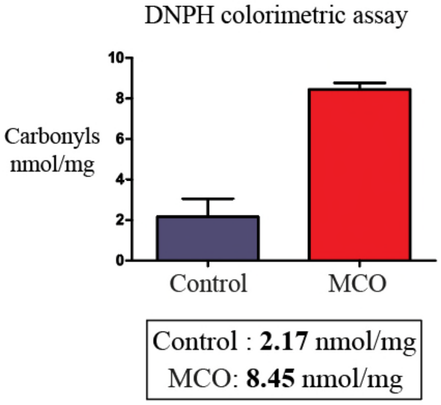

c

m/z	z	Peptide + Oxi-DNP modification	Error ppm	Score	Expect	Area in FMS
**614.7578**	2	CELAAAMK^{13}[+178.0127]R	-9.4	28.9	1.10E-04	1.47E+06
**752.3223**	2	GYS^{24}[+178.0127]LGNWVCAAK	-4.1	17	1.70E-03	2.89E+05
**768.3348**	2	GYSLGNW^{28}[+210.0025]VCAAK	19	17.1	2.30E-03	2.80E+06
**769.3386**	2	GYSLGNW^{28}[+212.0182]VCAAK	14	31.2	2.20E-04	3.69E+05
**803.8241**	2	FESNFNTQAT^{43}[+178.0127]NR	-14	22.7	1.90E-06	3.34E+05
**602.2157**	2	W^{62}[+210.0025]WCNDGR	18	19.7	7.10E-05	1.32E+07
**886.7518**	3	NLCNIP^{79}[+150.0178]CSALLSSDITASVNCAK	13	24.2	1.50E-03	6.71E+05
**901.4000**	3	NLCNIPCS^{81}[+194.0076]ALLSSDITASVNCAK	-4.2	28.4	4.10E-05	3.62E+05
**901.4000**	3	NLCNIPCSAL^{83}[+194.0076]LSSDITASVNCAK	-4.2	31.1	1.10E-06	3.62E+05
**896.0682**	3	NLCNIPCSALLS^{85}[+178.0127]SDITASVNCAK	-4.3	26	8.50E-07	2.87E+05
**943.4082**	2	I^{98}[+194.0076]VSDGNGM[+16]NAWVAWR	3	16.1	1.60E-04	1.63E+05
**943.4082**	2	IVS^{100}[+194.0076]DGNGM[+16]NAWVAWR	3	17.9	1.30E-04	2.85E+05
**943.3994**	2	IVSDGNGM[+16]NAW^{108}[+194.0076]VAWR	-6.4	23.3	3.30E-04	3.21E+06
**943.3994**	2	IVSDGNGMNAW^{108}[+210.0025]VAWR	-6.4	23.3	2.20E-04	3.21E+06
**951.3895**	2	IVSDGNGM[+16]NAW^{108}[+210.0025]VAWR	-14	21.6	3.10E-04	5.89E+05
**620.2711**	2	GTDVQ^{121}[+194.0076]AWIR	-12	18	6.00E-04	3.43E+05
**628.2641**	2	GTDVQAW^{123}[+210.0025]IR	-19	17.7	5.60E-05	2.08E+06
**620.2728**	2	GTDVQAW^{123}[+194.0076]IR	-9.5	24.2	6.00E-04	2.11E+05
**620.2728**	2	GTDVQAWI^{124}[+194.0076]R	-9.5	27.4	4.40E-05	1.42E+05

d

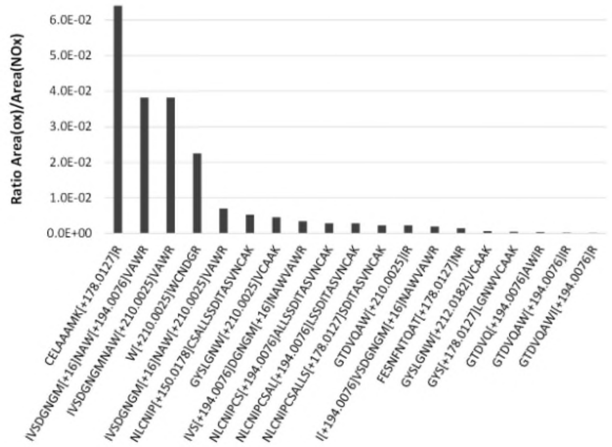

e

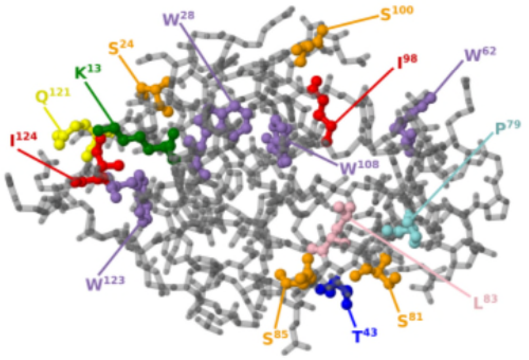

f

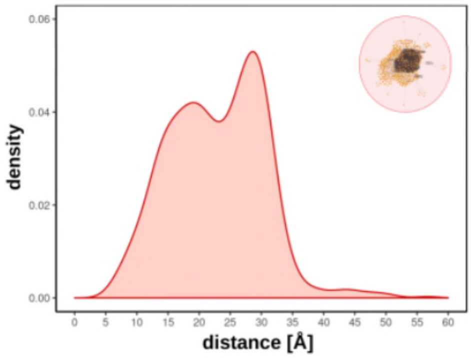

<u>Supplementary Figure S3:</u> **Characterization of oxidized lysozyme. a.** Carbonyl detection of lysozyme protein by 1D-WB. The non-oxidized control and oxidized protein were used in all experiments. The derivatization of the carbonyls was done using biotin-Ao probe and the detection of the carbonylated protein was done using streptavidin-Alexa Fluor700 conjugate. Protein loading was determined by Ponceau staining. The displayed images are representative of three replicates. Original and replicates of the blot are available in Supplementary Data 1 Fig. S8 **b.** Protein carbonyl content was determined using DNPH colorimetric assay. Quantification data for **a** and **b** are provided in the Supplementary Data 2 Figs. S14 and S15. **c** List of oxidized peptides identified in MCO Lysozyme sample using Protein Prospector. **d.** Ratio of oxidation of each peptide for Lysozyme. Peak area of the modified peptide in MCO sample, Area(Ox), divided by peak area of the corresponding non-modified peptide in the control sample, Area(NOx). **e.** 3D pdb structure of lysozyme (grey) with coloured residues found to be carbonylated according to c. **f.** Density of water molecules (800 H_2O) as a function of radial distance from Ao within the lowest energy structure of $Au_{15}SG_{12}$-Ao-Lysozyme.

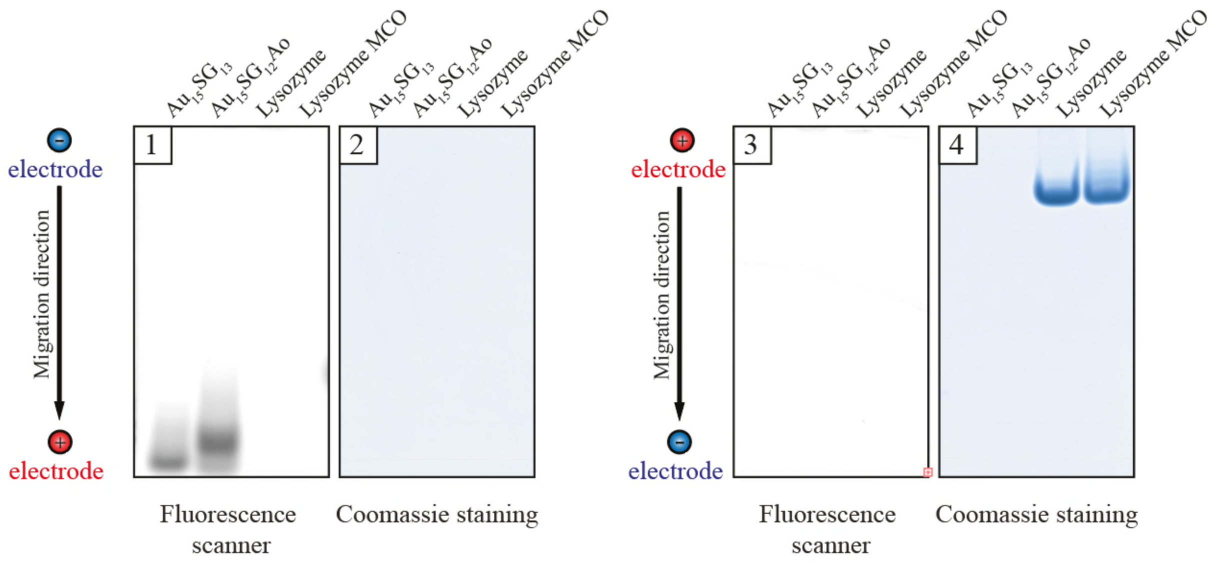

<u>Supplementary Figure S4:</u> Demonstration of the NCs and protein migration in this experimental setup. Scans of the four native gels. Gels 1 and 3 were dried and imaged using a fluorescence scanner. Gels 2 and 4 were stained using Coomassie staining and image were obtained using a gel scanner. NCs with and without functionalization and Lysozyme protein with and without oxidation were migrated to show their migration patterns. Original gels are provided in Supplementary Data 1 Fig. S13.
